# Inspection of Transparent Objects with Varying Light Scattering Using a Frangi Filter

**DOI:** 10.3390/jimaging7020027

**Published:** 2021-02-05

**Authors:** Dieter P. Gruber, Matthias Haselmann

**Affiliations:** Polymer Competence Center Leoben GmbH, 8700 Leoben, Austria; matthias.haselmann@pccl.at

**Keywords:** machine vision, surface inspection, anomaly detection, defect detection, Frangi filter, transparent components

## Abstract

This paper proposes a new machine vision method to test the quality of a semi-transparent automotive illuminant component. Difference images of Frangi filtered surface images are used to enhance defect-like image structures. In order to distinguish allowed structures from defective structures, morphological features are extracted and used for a nearest-neighbor-based anomaly score. In this way, it could be demonstrated that a segmentation of occurring defects is possible on transparent illuminant parts. The method turned out to be fast and accurate and is therefore also suited for in-production testing.

## 1. Introduction

Defect detection of freeform 3D-objects and surfaces, as they occur in optical illuminant elements of automotives and aircrafts is a challenging task. However, sales are dependent on the visual quality impression that products leave on potential customers. In particular, this applies to automobiles and consumer electronics, where the complexity of 3D-shaped components does not allow absolutely reliable manual quality assessment in the production. Therefore, the demand for machine vision solutions is persistently high.

The subjective nature of manual visual assessment of complicated surfaces combined with time pressure inevitably leads to errors. On the other hand, the absolute avoidance of defect part sales leads to a high number of rejected parts that are indeed acceptable. With automated surface inspection systems, attention must therefore be paid not only to reliably find clear defects but also to reliably ignore permitted part-to-part appearance variations. Various approaches have already been investigated for surface defect detection. The approaches depend not only on the surface itself but also on the defects to be detected. Inspection methods for gloss disturbances [1] differ, for example, from inspection methods for sink marks on surfaces [2,3], which is mainly due to the specialized multi-shot measurement methods for those type of defects. For defects that are visible on single surface images, there is a much wider range of investigated approaches, as examined in several survey articles [4,5,6]. Over the last years, an increasing proportion of methods are based on convolutional neural networks (CNNs) [7]. One problem, however, is that CNNs usually struggle to provide a solid performance with a small two-digit number of training images. Although there exist some approaches based on few-shot learning [8] or one-class learning [9], it is difficult to tweak the behavior of the inspection algorithm without new examples. In this context, approaches that do not rely on CNNs have an advantage.

**Figure 1:**
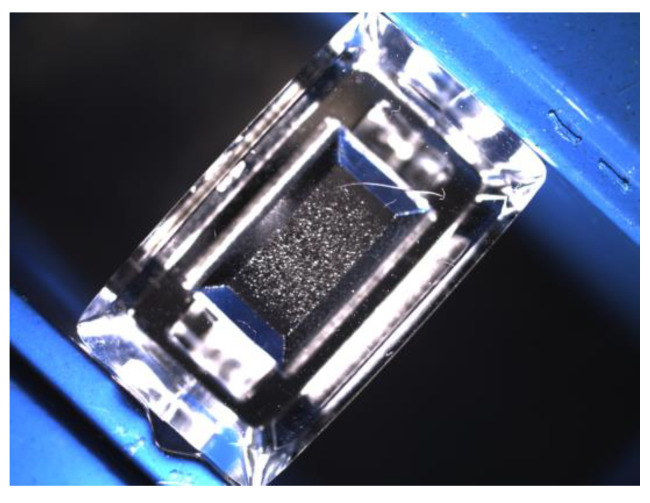
A transparent illuminant component with a defect (scratch).

**Figure 2:**
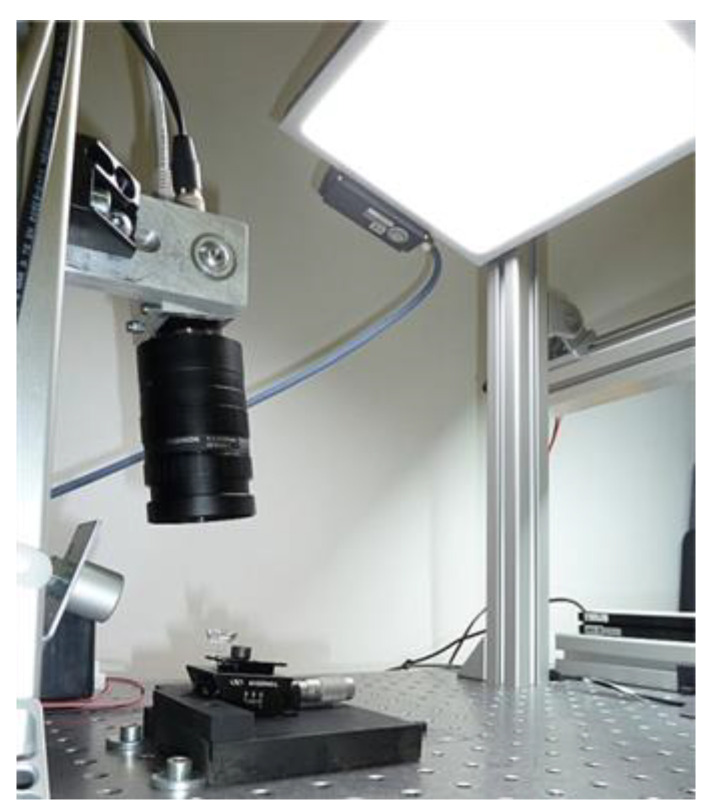
Image acquisition setup. The camera and an LED panel were positioned in such a way that the light specularly reflected by the component falls directly in the direction of the camera axis. This provided the best defect representation in the images.

In the automotive sector, transparent illuminant components are widely used. Figure 1 shows an example of a polymer illuminant part with a complicated light scattering structure and an obvious defect (scratch) in the upper half of the part. In order to detect defects on those parts, an inspection algorithm is proposed which is based on difference images of Frangi filtered surface images followed by a morphological feature extraction and the computation of a nearest-neighbor-based anomaly score for each detected structure in the image. The surface images of the investigated illuminant components were taken with the setup shown in Figure 2. Due to the optical lever, even tiny changes in the positioning of the part as well as in the illumination setup already cause a significant change in the surface appearance. For this reason, an implicit robustness of the inspection method against weak changes in the arrangement of regular structures was a substantial requirement. In this paper, it is demonstrated that with the proposed method, a segmentation of even very slightly visible defects is possible on the examined parts.

## 2. Theory—Frangi Filter

The filtering of second order local structures of an image is described e.g., by Chen and Hale [10] and Du et al. [11] with the aim to emphasize blood vessels in medical images. Early variants work on a fixed scale and therefore show problems in the recognition of structures that include a large size range. Multi-scale variants were performed by Sato et al. [12] and Lorenz et al. [13] and they were modified later by Frangi et al. [14]. The analysis of second order local image structures using its eigenvalues is advantageous since the main directions are represented by the eigenvalues themselves. The smallest and largest local curvature is obtained automatically and it is possible to amplify structures on a targeted scale. 

The local behavior of an image *I* in a point *x_0_* can be described by the Taylor series [14]:

(1) $$I\left(x_{0}+\delta x_{0},\sigma \right)\approx I_{x_{0},\sigma }+\frac{\partial }{\partial \delta x_{0}}I_{x_{0},\sigma }+\left(\frac{\partial }{\partial \delta x_{0}}\right)\left(\frac{\partial }{\partial \delta x_{0}}\right)I_{x_{0},\sigma }=I_{x_{0},\sigma }+\delta x_{0}^{T}\nabla_{x_{0},\sigma }+\delta x_{0}^{T}H_{x_{0},\sigma }\delta x_{0},$$

where ∇ is the gradient and *H* the hessian of the image in point *x*_0_. According to the linear scale-space theory, differentiation can be computed very efficiently by convoluting the image with the derivative of the 2-dimensional Gaussian [14]:

(2) $$\frac{\partial }{\partial x}I\left(x,\sigma \right)=\sigma^{\gamma }I\left(x\right)\frac{\partial }{\partial x}G\left(x,\sigma \right),\;\;G\left(x,\sigma \right)=\frac{1}{2\pi \sigma^{2}}\exp\left(-\frac{{\left\|x\right\|}^{2}}{2\sigma^{2}}\right).$$

The standard deviation *σ* of the Gaussian kernel function *G*(*x*) determines the scale of the amplified structures, while *γ* adjusts the filter responses across multiple scales [15].

**Figure 3:**
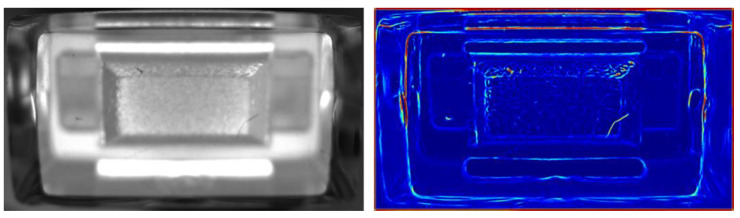
Registered image of an illuminant component (**left** side) and the response of the Frangi filter (**right** side).

Regarding the Hessian, the corresponding second order kernel function correlates well to the contrast between the regions inside and outside the interval [*x*_0_ − *σ*, *x*_0_ + *σ*] in direction of the derivative. Consequently, by analyzing the second order derivative in its principal directions, dark contrasted structures can be amplified depending on their shape. This is done first by computing the eigenvalues *λ* of the Hessian:

(3) $$H_{x_{0},\sigma }{û}_{\sigma ,k}=\lambda_{\sigma }{û}_{\sigma ,k}\Rightarrow \left(H_{x_{0},\sigma }-\lambda_{\sigma }U\right){û}_{\sigma ,k}=0,$$

where *U* is the identity matrix and *û_σ,k_* are the eigenvectors. The filter response is then computed according to the function:

(4) $$F_{x_{0},\sigma }=\{\begin{array}{c} 0,\;\;\lambda_{2}>0\\ \exp\left(-\frac{{\left(\lambda_{1}/\lambda_{2}\right)}^{2}}{2\beta^{2}}\right)\left[1-\exp\left(-\frac{{\left(\lambda_{1}+\lambda_{2}\right)}^{2}}{2c^{2}}\right)\right] \end{array}$$

where provided eigenvalues are in order |*λ*_1_| ≤ |*λ*_2_|. The first exponential factor for *λ*_2_ to be equal or lower than zero has its minimum for blob-like structures and responses increasingly to line-like structures controlled by the parameter *β*. The second factor—controlled by the parameter *c*—is a measure for second order structure [14], and suppresses image regions where no structure is present. The restriction of *λ*_2_ to be equal or lower than zero, guarantees that only dark contrasted structures are responded. In order to amplify bright contrasted structures as well, one has to repeat the filtering on inverted gray scale images. This type of filtering is suitable for surface inspection because occurring defects are accompanied by second order structures (see Figure 3). As expected, not only defective structures are amplified but also permitted ones. These two cases must be distinguished from each other. One method for this purpose is presented in the given article.

In addition to the Frangi filter, other filters such as the Sato filter [12], the Meijering neuriteness filter [16], and the hybrid Hessian filter [17] are in principle also suitable for the proposed approach. They show similar properties and have in common that they are used for detecting continuous ridges, such as vessels and tubes. Although the field of application of the mentioned filters is mainly in the medical image processing sector (e.g., amplification of blood vessels [12] and wrinkle detection [17]), they have also been used for surface defection tasks. For example, Zhang et al. [18] used a mean sampling method for fabric images, on which the Frangi filter was then used to enhance fabric defects. In the method proposed in this paper, it was never shown before in the field of the defect inspection of transparent components.

## 3. Methodology

**Figure 4:**
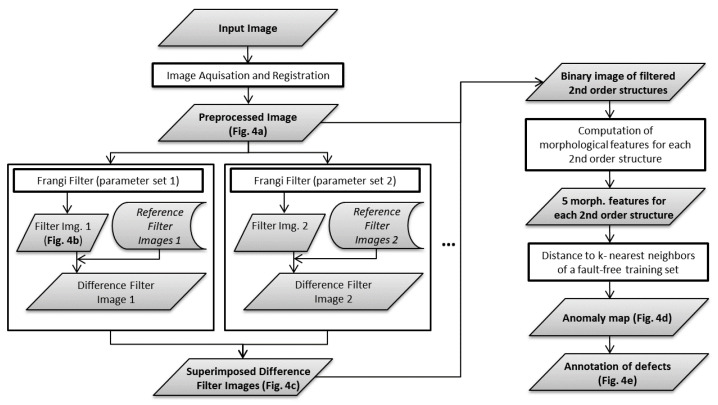
Flowchart of the proposed defect detection algorithm.

**Figure 5:**
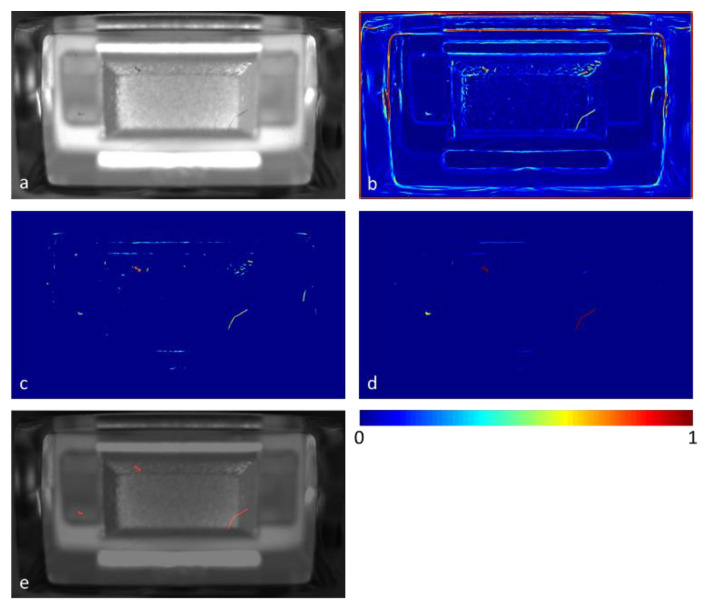
Inspection of a defective illuminant part with of the proposed inspection algorithm (interim results). (**a**) Shows the input image after registration, (**b**) the Frangi filtered image, and (**c**) the difference filter image based on the filtered input image and the filtered golden samples. The anomaly map (**d**) shows the anomaly score for every connected component. In (**e**), structures that are classified as defective according to a predefined threshold for the anomaly score are superposed on the input image. In the lower right, the scale bar of the false color images is depicted.

The proposed algorithm can be divided into five steps. A flow chart about the algorithm including its intermediate results is provided in Figure 4 and Figure 5. The first step (a) is to register the recorded images to maximize consistency between different images of the same type of part (see Figure 5a).

In step (b), the Frangi filter (see section Theory—Frangi Filter) is applied to the gray scale image to be inspected (see Figure 5b). The resulting filter image shows second order image structures, including occurring defects as well as normal, permitted structures. In step (c), the same filter is applied to a set of reference images that are defined as fault-free. The fixed reference filter images are superimposed by max-pooling and subtracted from the filter image to be inspected. Pixel values smaller than zero are clipped to zero. As a result, the majority of normal filter response (originating from permitted second order image structures) is removed in the resulting difference filter image, leaving structures that are well separated from each other. Step (b) and (c) can be repeated with different sets of Frangi filter parameters as well as on inverted gray scale images to amplify bright contrasted structures as well. All computed partial difference filter images are superimposed by max-pooling (see Figure 5c).

In step (d), the isolated image structures are considered as connected components (8-connectivity) and a morphological analysis on each of their binary counterparts is performed. This involves the extraction of 5 features (centroid position (2 features), orientation, area, and skeleton length) for each connected component. Then, an anomaly score is computed for each connected component based on the respective 5-dimensional feature vector. This is done by one-class learning on a set of fault-free samples that is different from the set of fault-free samples used in step (c). The anomaly score for each inspected connected component is then obtained by measuring the Euclidian distance to its respective nearest neighbor in the fault-free training set (see Figure 5c). Finally, in step (e), a binary classification between defect and allowed structures can be done by defining a threshold for the anomaly score (see Figure 5e).

## 4. Experimental Results

For testing the proposed algorithm on the described illuminant components, five fault-free parts were used as a basis for the filtered difference images (step c). In addition, ten fault-free parts were used in the final step as training set for the nearest-neighbor-based anomaly score. By this means, a total of 500 connected components—defined as fault-free—could be extracted as training instances for the nearest neighbor algorithm. For the filtering in step b and c only one parameter set—sigma = 3, beta = 1.5, c = 5—was used. However, it was applied on both the original gray scale image and its inverted version. While the former responds to dark contrasted second order structures, the latter responds to bright contrasted ones.

**Figure 6:**
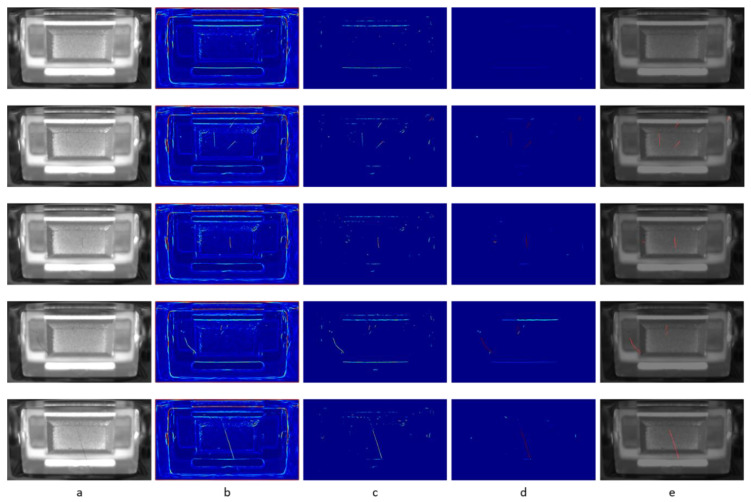
Five examples of inspected parts. Column (**a**) shows the registered input images to the pipeline. Note the slight variations of the surface appearance between the parts regardless of occurring defects. Column (**b**) shows the Frangi filtered images, (**c**) the difference filter images, (**d**) the anomaly score of the connected component in the difference filter images, and (**e**) the input image overlaid with the detected defects (red).

Since there were too few test parts available for obtaining a reliable statistic about the proposed method, the results are demonstrated on individual examples (see Figure 6). Apparently, the distinction between defects and allowed second order structures is possible with the proposed method, despite part-to-part appearance variations.

Without filtering the raw images, the part-to-part variations make the difference images quite useless for detecting anomalies, since even small variations cause strong responses in the difference image. With the application of the Frangi filter, however, crucial image structures are not only enhanced but also strongly blurred, so that a small offset between similar structures in two images does not produce a large signal in the difference image. In contrast, structures resulting from a defect produce a large signal. In addition to the Frangi filter, other filters with similar properties [12,16,17] are apparently also suitable for the proposed approach. Since in the proposed approach the filtering is only one step out of 5 steps and the algorithm worked well on the few parts available for testing, it was decided not to substitute the Frangi filter with other filters.

## 5. Conclusions

In this paper, a defect detection algorithm for surface images is proposed, in which a Frangi filter is used to amplify second order image structures. The majority of the filter response is then removed by using equally filtered reference images that are defined as fault-free. The resulting isolated second order structures, including potential defects, are then morphologically analyzed with the aim to extract a small feature vector for each structure. In the last step, for each feature vector, an anomaly score is computed by measuring the distance to its nearest neighbor originating from a fault-free training set. By this means, it could be exemplarily demonstrated that defect segmentation is possible despite significant part-to-part appearance variations. The method turned out to be fast and accurate and therefore it is suited for in-production testing.

## Data Availability

The data are not publicly available due to the compliance rules of involved organizations.

## References

[1] Gruber D.P., Buder-Stroisznigg M., Wallner G., Strauß B., Jandel L., Lang R.W. (2012). Characterization of gloss properties of differently treated polymer coating surfaces by surface clarity measurement methodology. Appl. Opt..

[2] Gruber D.P., Berger G., Pacher G., Friesenbichler W. (2011). Novel approach to the measurement of the visual perceptibility of sink marks on injection molding parts. Polym. Test..

[3] Gruber D.P., Macher J., Haba D., Berger G.R., Pacher G., Friesenbichler W. (2014). Measurement of the visual perceptibility of sink marks on injection molding parts by a new fast processing model. Polym. Test..

[4] Xie X. (2008). A review of recent advances in surface defect detection using texture analysis techniques. Electron. Lett. Comput. Vis. Image Anal..

[5] Chandola V., Banerjee A., Kumar V. (2009). Anomaly Detection: A Survey. ACM Comput. Surv..

[6] Pimentel M.A.F., Clifton D.A., Clifton L., Tarassenko L. (2014). A review of novelty detection. Signal Process..

[7] Chalapathy R., Chawla S. (2019). Deep Learning for Anomaly Detection—A Survey. arXiv.

[8] Qianwen L., Yonghong S. (2019). Few-shot Learning Combine Attention Mechanism-Based Defect Detection in Bar Surface. ISIJ Int..

[9] Haselmann M., Gruber D.P., Tabatabai P. Anomaly Detection Using Deep Learning Based Image Completion. Proceedings of the 2018 17th IEEE International Conference on Machine Learning and Applications (ICMLA).

[10] Chen H., Hale J. (1995). An algorithm for MR angiography image enhancement. Magn. Reson. Med..

[11] Du Y.P., Parker D.L., Davis W.L. (1995). Vessel enhancement filtering in three-dimensional MR angiography. J. Magn. Reson. Imaging JMRI.

[12] Sato Y., Nakajima S., Shiraga N., Atsumi H., Yoshida S., Koller T., Gerig G., Kikinis R. (1998). Three-dimensional multi-scale line filter for segmentation and visualization of curvilinear structures in medical images. Med. Image Anal..

[13] Lorenz C., Carlsen I.-C., Buzug T.M., Fassnacht C., Weese J., Goos G., Hartmanis J., van Leeuwen J., Troccaz J., Grimson E., Mösges R. (1997). Multi-scale line segmentation with automatic estimation of width, contrast and tangential direction in 2D and 3D medical images. CVRMed-MRCAS'97.

[14] Frangi A.F., Niessen W.J., Vincken K.L., Viergever M.A., Wells W.M., Colchester A., Delp S. (1998). Multiscale vessel enhancement filtering. Medical Image Computing and Computer-Assisted Intervention—MICCAI’98.

[15] Koller T.M., Gerig G., Szekely G., Dettwiler D. Multiscale detection of curvilinear structures in 2-D and 3-D image data. Proceedings of the IEEE International Conference on Computer Vision.

[16] Meijering E., Jacob M., Sarria J.C., Steiner P., Hirling H., Unser M. (2004). Design and validation of a tool for neurite tracing and analysis in fluorescence microscopy images. Cytom. Part A.

[17] Ng C.C., Yap M.H., Costen N., Li B. (2014). Automatic wrinkle detection using hybrid Hessian filter. Asian Conference on Computer Vision.

[18] Zhang H.H., Li R.-Z., Li P.-F., Jing J.-F., Zhao J. (2015). Fabric defect detection based on Frangi filter. Wool Text. J..

